# Electrochemical Methodologies for Investigating the Antioxidant Potential of Plant and Fruit Extracts: A Review

**DOI:** 10.3390/antiox11061205

**Published:** 2022-06-20

**Authors:** Mir Waqas Alam, Jawayria Najeeb, Sumaira Naeem, Sheikh Muhammad Usman, Insha Nahvi, Fai Alismail, Alaaedeen Abuzir, Mohd Farhan, Allah Nawaz

**Affiliations:** 1 Al Bilad Bank Scholarly Chair for Food Security in Saudi Arabia, The Deanship of Scientific Research, The Vice Presidency for Graduate Studies and Scientific Research, King Faisal University, Al-Ahsa 31982, Saudi Arabia; iahmad@kfu.edu.sa (I.N.); 218019406@student.kfu.edu.sa (F.A.); aabuzir@kfu.edu.sa (A.A.); mfarhan@kfu.edu.sa (M.F.); 2 Department of Physics, College of Science, King Faisal University, Al-Ahsa 31982, Saudi Arabia; 3 Department of Chemistry, University of Gujrat, Gujrat 50700, Pakistan; jawayria@uog.edu.pk; 4 Hunza Sugar Mills Private Limited (Distillery Division), Lahore 54000, Pakistan; shmusmanjaved@gmail.com; 5 Department of Basic Sciences, Preparatory Year Deanship, King Faisal University, Al-Ahsa 31982, Saudi Arabia; 6 Department of Biomedical Engineering, College of Engineering, King Faisal University, Al-Ahsa 31982, Saudi Arabia; 7 Department of Molecular and Medical Pharmacology, Faculty of Medicine, University of Toyama, 2630 Sugitani, Toyama-shi 930-0194, Japan; nawaz@med.u-toyama.ac.jp

**Keywords:** antioxidant capacity, antioxidants, plant extract, spectrophotometric technique, cyclic voltammetry

## Abstract

In recent years, the growing research interests in the applications of plant and fruit extracts (synthetic/stabilization materials for the nanomaterials, medicinal applications, functional foods, and nutraceuticals) have led to the development of new analytical techniques to be utilized for identifying numerous properties of these extracts. One of the main properties essential for the applicability of these plant extracts is the antioxidant capacity (AOC) that is conventionally determined by spectrophotometric techniques. Nowadays, electrochemical methodologies are emerging as alternative tools for quantifying this particular property of the extract. These methodologies address numerous drawbacks of the conventional spectroscopic approach, such as the utilization of expensive and hazardous solvents, extensive sample pre-treatment requirements, long reaction times, low sensitivity, etc. The electrochemical methodologies discussed in this review include cyclic voltammetry (CV), square wave voltammetry (SWV), differential pulse voltammetry (DPV), and chronoamperometry (CAP). This review presents a critical comparison between both the conventional and electrochemical approaches for the quantification of the parameter of AOC and discusses the numerous applications of the obtained bioextracts based on the AOC parameter.

## 1. Introduction

**Figure 1:**
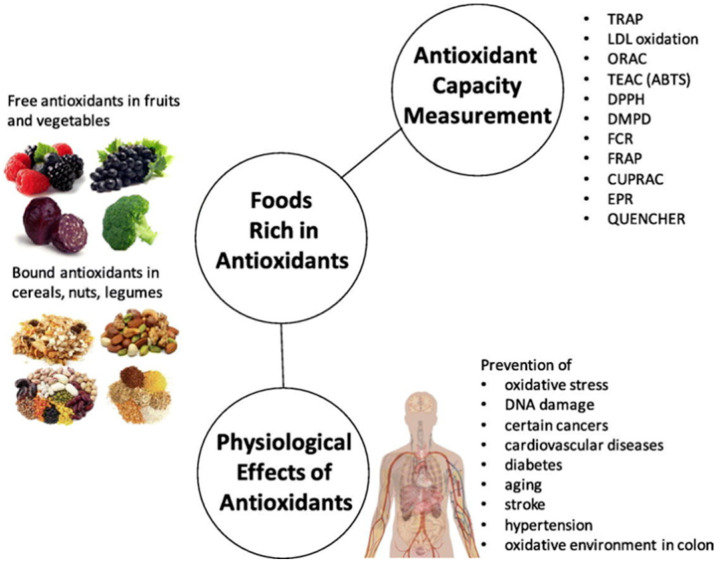
Evolution of food antioxidants as a core topic of food science. Figure adapted with permission from Ref. [8]. Elsevier.

During the metabolic conversions of food into the energy, excess amounts of the free radicals are constantly generated in the human body. These unstable and highly reactive free radical species are a constant threat to the constituents of the cells (particularly the genetic materials) and destroy the constituents via numerous destructive mechanisms [1]. Therefore, the modulation of the concentration of these free radical species is an essential task [2]. Though oxidative metabolism plays an essential role in the survival of our cells, the production of free radicals (along with some other reactive oxygen species) during this process still causes various oxidative changes [3]. However, we are not defenseless against these free radicals and their relentless attack. This is attributed to the presence of certain molecules in our bodies called antioxidants. Antioxidants operate in two ways in order to address the issue of the oxidative stress in the human body: (1) the antioxidants scavenge/quench these reactive oxygen species (ROS) or reactive nitrogen species (RNS) by chain-breaking mechanisms (these antioxidants are called primary antioxidants) [4]; and (2) the antioxidants that suppress the generation of the oxidation promoters (such as singlet oxygen, metal ions, and pro-oxidation enzymes, etc.) by chelation mechanisms (these antioxidants are called secondary or preventive antioxidants) [5]. The most familiar out of thousands of such antioxidant substances are Vitamin C, Vitamin E, beta carotenes, many related carotenoids, and minerals like selenium and manganese, etc. [6]. Antioxidants hold great interest for pharmacists and biochemists because of their capability to moderate the damages caused by RNS, ROS, or even chlorine-like reactive species [7]. The importance of antioxidants is highlighted in the food preservation applications as presented in Figure 1.

Growing interest has been recently observed in developing cost-effective and reliable techniques for screening and quantifying the antioxidants present in various biological/biogenic samples. The quantification of the antioxidant potential is done by using the parameter of the antioxidant capacity (AOC), which represents the concentration or number of moles of the specific free radical species scavenged by a particular antioxidant present in the sample [9]. The AOC parameter is a thermodynamic parameter and is found to be connected with the equilibrium constant of the process (scavenging reaction of antioxidant) [10]. Though a number of methodologies have been reported for estimating the AOC of the antioxidants, identifying the direct-action mechanism of antioxidants still remains a challenge in the field of free radical chemistry [10]. Antioxidant behavior is quantified using the two broad-term methodologies that reflect the focus on activity in foods (first category) and bioactivity in humans (second category). The category focusing on food systems involves the assessment of antioxidant efficacy, especially in fruits, vegetables and beverages so that a clear observation of their dietary burden and in vivo activity could be reported. As far as the antioxidant status in humans is concerned, there has been reported an obvious observation regarding an imbalance between the reactive oxygen species and defense/repair mechanisms in vivo [3,11]. Understanding both quantification methodologies (usually termed as antioxidant assays) is essential in developing a clear picture of the working of antioxidants.

**Figure 2:**
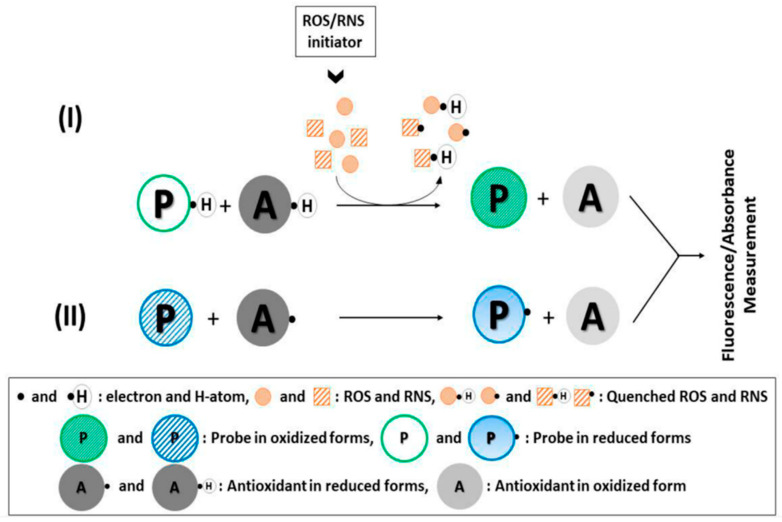
(I) Direct (competitive) antioxidant assay involving a fluorogenic or chromogenic 1263 probe and biologically relevant ROS/RNS; (II) indirect (noncompetitive) antioxidant assay in 1264 probe where the physiological redox reactions (i.e., oxidant–antioxidant interactions) are simulated on 1265, an artificial probe without biologically relevant ROS/RNS. Figure adapted with permission from Ref. [12]. Copyright 2019 American Chemical Society.

The antioxidant assays can be broadly categorized into two classes of direct antioxidant assays and indirect antioxidant assays [12]. The first category of direct assays is the competitive technique, and the added probe, as well as the antioxidants present in the sample both competes with each other to attack the free radical/free radical initiator. This makes these direct assays a little less accurate technique to detect the AOC [11]. The indirect antioxidant assays are non-competitive tests where an artificial probe (an oxidant) is added to the sample to observe the impact of the antioxidant action on this probe. The attack of antioxidant molecules generates structural changes in the added probe, which is measured by spectroscopic, fluorescence, electrochemical, or other methodologies. The indirect assays are more common and provide more accurate results in comparison to the direct assays owing to their non-competitive nature [12]. Figure 2 shows the schematics of the mechanism of direct or indirect assays used in this regard. Furthermore, it should also be mentioned here that the free radicals are scavenged by utilizing two mechanisms, including the electron transfer (ET) and hydrogen atom transfer (HAT) mechanism. In the ET mechanism, the compound of interest is reduced by the transference of one or more electrons from antioxidants to the unstable species, while in the HAT mechanism, the compound of interest is reduced by using the transference of the hydrogen atom to the probe [12].

In this review article, critical comparisons of numerous antioxidant assays utilized for the estimation of AOC in the plant/fruit extracts are presented. The first section provides a brief description of the fundamentals of antioxidants. The second section represents a summary of the conventional methodologies, while the third section provides a detailed analysis of the electrochemical techniques utilized for the estimation of AOC in the biogenic extracts. Some peculiar case studies explaining the applicability of the electrochemical techniques for the estimation of AOC are summarized in the fourth section. The last section details the conclusion and future prospects associated with this particular research domain of electrochemical techniques used for the determination of AOC.

## 2. Conventional Assays/Methods for Determination of AOC

**Figure 3:**
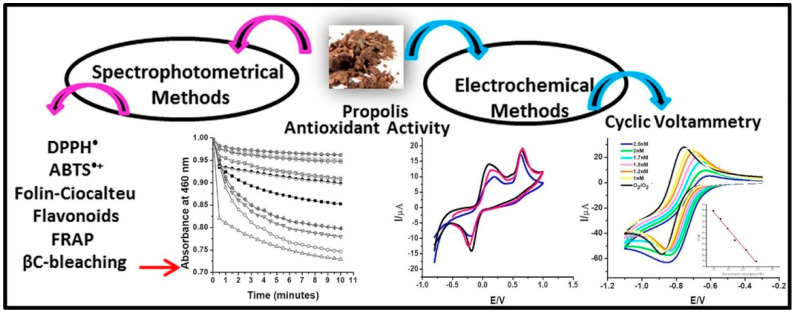
Comparative study of different methodologies for the determination of the antioxidant activity of *Venezuelan propolis*. Figure adapted with permission from Ref. [15]. Copyright 2020 Elsevier.

Many analytical techniques have been developed for the extraction, separation, identification and quantification of antioxidant compounds by using various conventional methodologies [13]. The classification of the numerous antioxidant assays utilized for the determination of AOC is presented in Table 1. AOC is either expressed in the form of equivalents of the selected reference antioxidants (particularly gallic acid and Trolox) in the case of radical/ROS-based scavenging assay or inhibition potential of the antioxidant against the oxidation of the added probe [14]. The oxidation of the added probe is determined by using analytical techniques such as fluorimetric, spectrophotometric, cyclic voltammetry, amperometric methods, and chemiluminescent, etc. [13]. The electrochemical methods fall under the category of non-radical/potential-based antioxidant assays and are not discussed in Table 1. The electrochemical methodologies are discussed in detail in the next section. A comparative study of different methodologies for the determination of the antioxidant activity of Venezuelan propolis, as an example, has been shown in Figure 3.

Advanced spectrometric methods have also been widely documented for analyzing the antioxidant properties of reported natural antioxidants found in the biogenic samples. However, these methodologies exhibit strong dependence on parameters such as optimization of temperature, time, concentration, and other physicochemical characterization of antioxidant substances, which is needed to be crucially considered before experimentation [27]. High-performance liquid chromatography has been primarily used for the separation, identification, and quantification of nutraceutical components for many years. However, mass spectrometry, when coupled with liquid chromatography, has recently been introduced as a detection method for many such investigations with even lower detection limits [28]. The summary of some case studies documenting the use of conventional and electrochemical detection methodologies used for the identification of AOC in natural and synthetic antioxidants, along with their applications, are provided in Table 2.

## 3. Electrochemical Methodologies

In recent years, electrochemical methodologies have emerged as the competing technique against the antioxidants for the determination of AOC in biogenic samples [41]. The increase in the applicability of these techniques for AOC determination is attributed to the fact that extremely low molecular weight anti-oxidants can be easily detected and screened by using these techniques. The electrochemical methods operate by calculating the total reducing power of the sample, which represents the ability of certain antioxidants to donate/accept electrons in a redox (oxidation–reduction) environment [42]. Electrochemical methods are considered very sensitive and rapid methodologies for the determination of antioxidant potential in both stationary as well as dynamic flow systems [43]. Moreover, it should be made clear that most electrochemical techniques provide the information regarding the total antioxidant capacity (TAC) values of the antioxidants, which represents the cumulative impact of all the antioxidants present in the sample in contrast to the determination of the individual antioxidant potentials measured via a parameter of AOC. Electrochemical techniques involved in evaluating the antioxidant potential of biogenic samples are discussed in detail in the subsection [44].

Electrochemical techniques address a few major drawbacks associated with chromatographic and spectroscopic techniques, such as long sample preparation times, use of hazardous solvents, long analysis time, undefined reaction time and expensive setups. Furthermore, the sensitivity and reproducibility of the electrochemical methods is also quite high in comparison to the above-mentioned conventional antioxidant assays. Stationary systems are known for their suitability in quantifying only a limited amount of analytes, but the study of simple as well as more complicated biomolecules in dynamic systems can also be easily conducted by using these methodologies [45]. A carbon working electrode can be used in the case of both fluid and solid systems. Carbon paste electrodes, printed carbon electrodes, paraffin, and silicon oils modified carbonaceous materials (carbon pastes) are also used for a similar purpose [42]. Sometimes, the addition of inorganic/organic nanomaterials is also done to increase the selectivity or specificity of these electrodes. These modified electrodes perform quite well when used in voltammetry, amperometry and cyclic voltammetry techniques [42,46].

### 3.1. Cyclic Voltammetry (CV)

**Figure 4:**
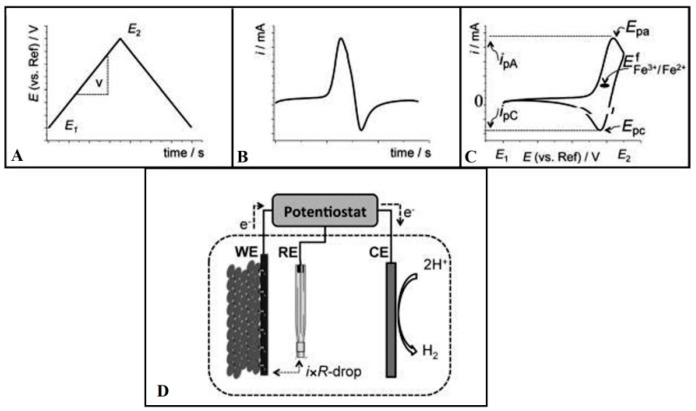
In a CV, (**A**) the potential (with respect to RE) is applied to the WE. The potential start at the lowest potential value (E_1_), gains a maximum potential value (E_2_) and then is reduced back to the lowest potential value (E_1_); (**B**) The current generated owing to this potential change is plotted against time; and (**C**) The experimental CV setup indicating RE, CE, WE, and (**D**) A three-electrode potentiostatic experimental setup for anodic biofilm. Figure adapted with permission from Ref. [47]. Copyright 2012 John Wiley and Sons.

The CV is one of the most exploited electrochemical techniques for the determination of TAC in biogenic extracts. The CV comprises a stationary working electrode (WE) which is linearly ramped with respect to the time in a triangular waveform (i.e., the potential is increased from the lowest to the maximum value and after attaining the maximum potential, the potential is reversed to again attain the lowest value) [47]. Besides the WE, two other electrodes called reference electrodes (RE) and auxiliary/counter electrode (CE) is also part of the CV setup. The galvanostat/potentiostat is attached with the CV assembly that records the current values attained owing to the redox reactions occurring in the medium and the current versus potential graph is plotted for the understudy sample [48]. The typical setup utilized for CV, as well as the utilized waveform and typical voltammogram shape, is presented in Figure 4.

**Figure 5:**
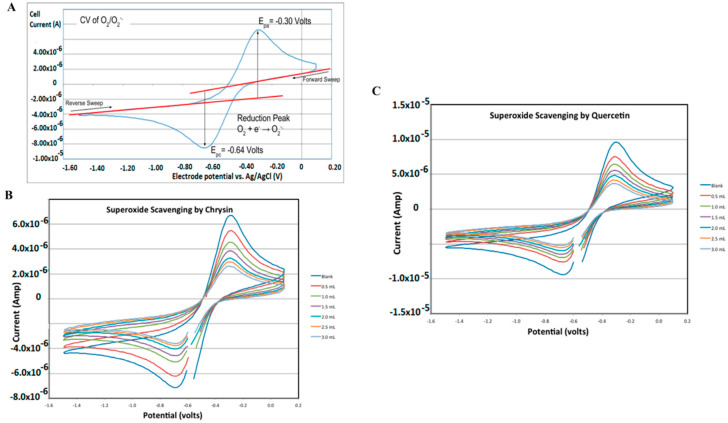
(**A**) CV voltammogram for superoxide radical ion; (**B**) Decrease in current values representing the superoxide radical ions in the presence of Chrysin; and (**C**) Decrease in current values representing the superoxide radical ions in the presence of Quercetin. Figure adapted with permission from Ref. [54]. Copy Right 2019 MDPI under Creative Commons Attribution Licence (CC BY 4.0).

The reversible system is represented by the equation O+ne↔R where O represents the oxidized form and R represents the reduced form. The typical CV voltammogram attained owing to the forward and reversed sweep in the case of superoxide radical is presented in Figure 5. As discussed previously, antioxidant scavenges the superoxide radicals, which results in a decrease in the current values observed in the presence of chrysin and quercetin (presented in Figure 5). This is a technique used to collect qualitative information while measuring the current response of any redox-active solution, such as the presence of intermediates in redox reactions to linearly cycled potential sweep between two or more set values [48]. Furthermore, this gives information about the characterization of redox systems, the number of redox states, stability of these states, and electron transfer kinetics of the redox reactions taking place in the medium [48,49]. The variation in the parameters of peak current (I_p_) and peak potential (E_p_) are the main tools utilized as a means to calculate the TAC value of antioxidants. It is used for determining the antioxidant activity of food, polyphenolic compounds [50], clinical samples [51], and pathological processes and infectious diseases [52,53].

### 3.2. Differential Pulse Voltammetry (DPV)

**Figure 6:**
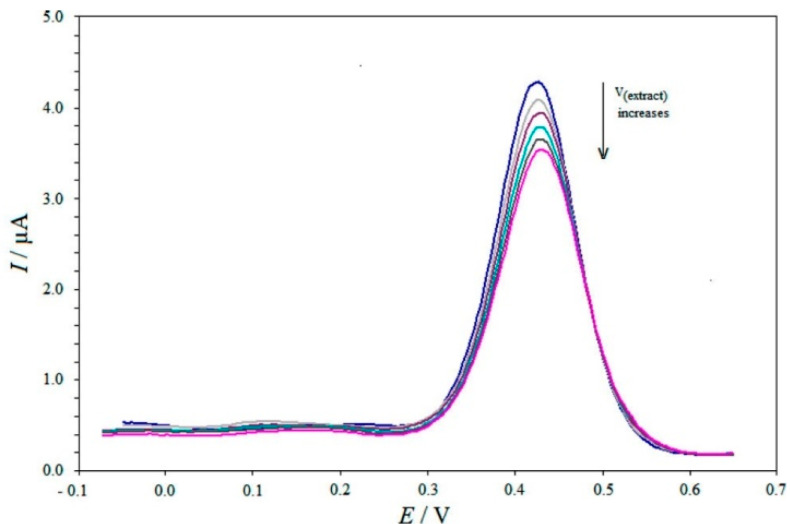
DPV voltammogram representing the reduction in the peak current owing to the presence of the antioxidants in the herbal tea extract. Figure adapted with permission from Ref. [58]. Copyright 2014, American Chemical Society.

The quantitative chemical analysis, kinetics, thermodynamics and mechanisms of chemical reactions have also been studied by another electrochemical technique of DPV [55]. The DPV technique superimposes the fixed-magnitude pulses on a linear potential ramp. The potential-time curve acquired in the DPV technique is generated by recording the response current, which was sampled twice and the current difference is plotted against the potential to acquire the DPV voltammogram [56]. These measurements are advantageous because the effect of the charging current can be minimized, resulting in increased sensitivity in current measurements [57]. Figure 6 represents the DPV technique where the current peak corresponds to the concentration of cupric neocuproine complex (Cu(Nc)_2_^2+^) and the addition of herbal tea extracts containing the antioxidants reduces this complex and consequently, peak current values are reduced. This reduction is utilized as a tool to measure the TAC of the antioxidants present in the tea extract [58]. Another important point is the extracted faradaic current which results in analyzing the electrode reactions more precisely [59]. It is also used effectively to measure the antioxidant capacity of food [60], plant extracts [61], and polyphenolic compounds such as procyanides and catechins from cone extracts which showed significant ability to reduce oxidative stress, scavenge free radicals, and transitions metal ions as well [62,63,64].

### 3.3. Square Wave Voltammetry (SWV)

**Figure 7:**
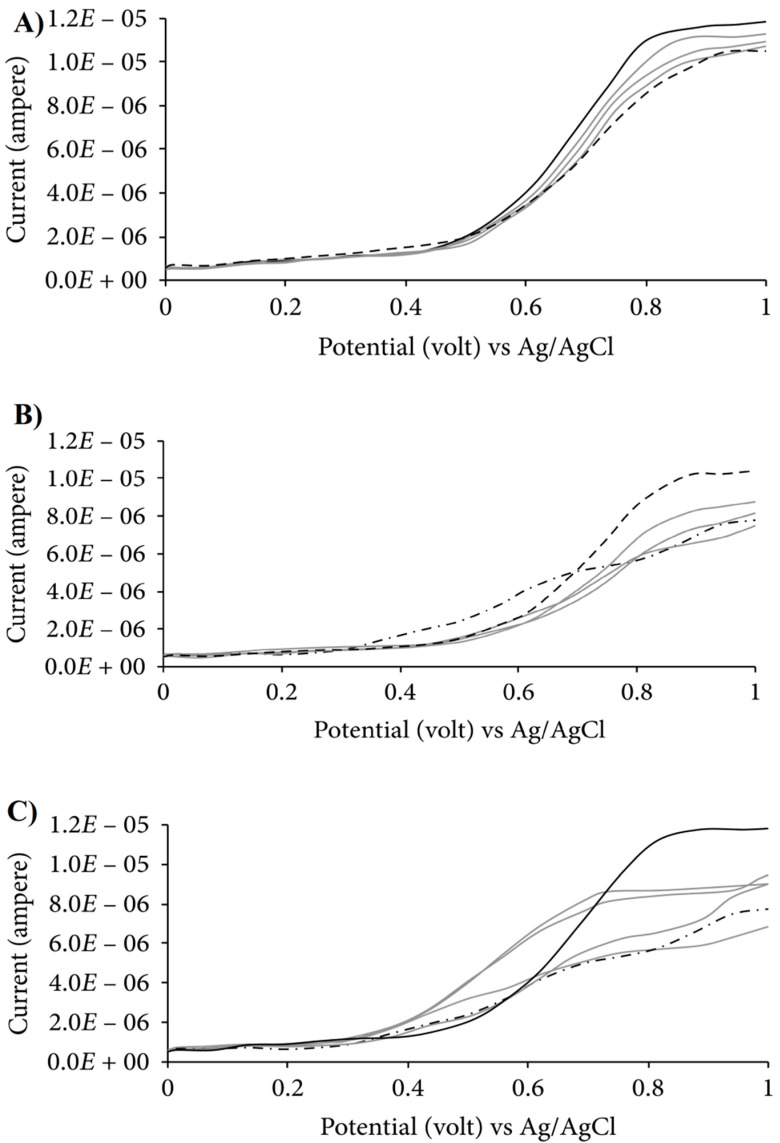
SWV voltammogram of binary mixture of extracts. (**A**) Blueberry and lemon; (**B**) blueberry and grape; (**C**) lemon and grape (where blueberry, lemon and grape extract are represented by a solid line, even dashed line and uneven dashed line, respectively, while gray lines represent the binary mixtures of the extracts in different ratios). Figure adapted with permission from Ref. [68]. Copyright 2019 Hindawi under Creative Commons Attribution Licence (CC BY 4.0).

This pulsed voltammetry technique uses a potential waveform (where the entire symmetrical potential square wave is superimposed on the staircase potential waveform) that is implemented on the WE [65]. The typical voltammogram acquired in the SWV is presented in Figure 7. As observed in the case of an electrochemical technique, the voltammograms of the understudy compound exhibit peak/peaks that correspond to the numerous redox processes that analytes experiences in the medium. The peak potential/potentials (potential at the maxima of the peak) also provide essential information regarding the tendency of the antioxidants in order to perform the ET reactions. Moreover, the antioxidants display the peaks at the lower values of oxidation potential, which displays the tendency of the antioxidants to be oxidized [66]. In comparison to the linear sweep voltammetry, the SWV is more sensitive and has a more extended dynamic range with lower detection limits. This technique was used to identify and quantify the synthetic antioxidants tert-butylhydroquinone (TBHQ) and butylated hydroxy anisole (BHA) in the presence of the cationic surfactant CPB using a carbon black electrode and analysis of food samples and biodiesel, and also in tea samples [67].

### 3.4. Amperometric Measurements (Chronoamperometry)

**Figure 8:**
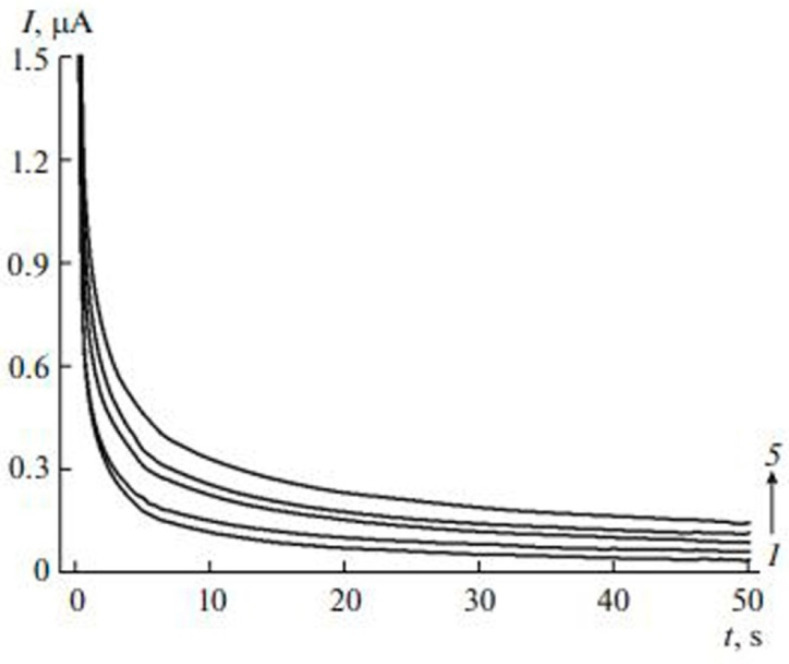
Chronoamperograms of (1) phosphate buffer, (2) blank, (3) semi-fermented tea leaves, (4) white tea leaves, and (5) green tea leaves. Figure adapted with permission from Ref. [72]. Copyright 2017 Springer Nature.

Chronoamperometry (CA) technique utilizes the stepping of the potential from the potential value at which there is no faradic reaction to the potential value and where the dose of electroactive species at the WE is essentially zero [69]. The variation in the response current is documented with respect to time to generate the CA potential-time graph. The typical chronoamperogram acquired in the case of the extracts of the tea leaves was performed by using the WE of glassy carbon electrode (GCE) that is modified with carbon nanotubes (MWCNTs) and electropolymerized quercetin (EPQ) is presented in Figure 8. Various antioxidant compounds have been analyzed for their antioxidant activity using amperometric techniques [70]. Disposable polyester screen-printed graphite macroelectrodes have been used coupled with a batch injection cell for measurement of consumption of DPPH. This technique measures electric current from oxidation of a substance such as food samples when studied on the surface of a working electrode at some fixed voltage potential using amperometric detectors [71].

## 4. Applications of the Electrochemical Methods for the AOC

Different case studies utilizing the electrochemical methods as a means for the determination of AOC are discussed in the subsection below [69,70,71,72]. These particular case studies are classified on the basis of the origin and utility of the biomass extract for this review.

### 4.1. AOC Determination of the Fruit’s Extract

A literature survey indicated that among the utilized electrochemical techniques, CV is the most abundantly exploited electrochemical technique used for the determination of the AOC in the fruit’s extract [73]. Antioxidants having moderately low-molecular weight have been particularly documented to be determined with quite an ease via this technique [74]. Zeliǹska et al. [75] examined 11 randomly selected samples of apple cultivars and used the techniques of CV for documenting the AOC, reduction, and chelating potential of the apple’s (peel and flesh) extract. The parameter of first anodic peak potential (E_pa_) was utilized to investigate the reduction potential of the phytochemicals present in the extract. The E_pa_ values of 0.815 V, 0.759 V, 0.618 V, 0.571 V, 0.515 V, 0.512 V, 0.511 V, 0.391 V, 0.390 V, 0.339 V and 0.334 V were recorded for the phytochemicals of phloretin, phloridzin, cyanidin 3-galactoside, catechin, quercetin 3-rhamnoside, quercetin 3-arabinoside, quercetin 3-glucoside, chlorogenic acid, rutin, epicatechin, and quercetin, respectively. The higher E_pa_ values are attributed to the lower reducing activity of the phytochemicals and these results are translated into the high to moderate antioxidant power. Area under the current wave was also utilized to detect the order of the AOC potential. Comparison and correlation studies revealed that the results acquired from CV technique were found to be most compatible with Ferric reducing antioxidant power (FRAP) assay among all other spectroscopic and chromatographic techniques. The literature survey further indicated that the peak current, the area under the current curve, and peak potential are the most important parameters for studying the AOC of the extract [76,77]. The peak potential of the acquired phytochemicals correlates with the type of the reducing agent utilized [78]. The lower oxidation potential values were found to be associated with the enhanced strength of particular phytochemicals for the electron donation and, therefore, directly provide information regarding the AOC of the molecule [79]. The Randles–Sevcik equation (discussed in the above equation) also provides a means to access the antioxidant dose present in the extract via the value of the peak current ($I_{p}$).

Jara-Palacios et al. [80] utilized the AOC values of the grape pomace (a solid residue composed of approximately 20% of total solid weight of the fruit of the grape, acquired as waste from the grape juice or wine industries) by using the CV technique and thiobarbituric acid reactive substances (TBARS) assay. The TBARS assay is a measure of inhibition of lipid peroxidation (the series of the particular chemical reactions responsible for the oxidative degradation of the lipids molecules). This inhibition is done owing to the presence of the antioxidants in the medium as peroxide molecules are scavenged by these molecules. Apart from identifying the AOC via CV, the electrochemical data was also used to study the by-products of these antioxidant reactions [80]. De Macêdo et al. [73] synthesized a laccase-based biosensor (LLB) and used it for the detection of antioxidants in the extracts of acerola, cranberry, camu-camu, and açai red fruits. The electroanalytical DPV, CV and SWV techniques were also used for this purpose and the parameter of the electrochemical index (EI, presented in Equation (1)) was evaluated for studying the antioxidants:

(1) $$\mathrm{El}=\frac{I_{\mathrm{pa}1}}{E_{\mathrm{pa}1}}+\frac{I_{\mathrm{pa}2}}{E_{\mathrm{pa}2}}+\ldots +\frac{I_{\mathrm{pan}}}{E_{\mathrm{pan}}}$$

where the I_pa1_ to I_pan_ and E_pa1_ to E_pan_ represent the anodic current and anodic potential of each successive anodic peak from 1 to n. The LLB and EI both represented the same order for the AOC potential in different varieties of the red fruits, i.e., camu-camu > cranberry > açai = acerola. However, the order of AOC potential documented by the DPPH assay did not relate to the LLB and electrochemical techniques, indicating that further research is still needed in this domain to fully comprehend the usefulness of the electrochemical techniques for measuring the AOC values [73]. The electrochemical techniques were also utilized for investigating the AOC values in the extracts of the following edible nutraceuticals, including apricot, black currant, grape pomace (by CV) [64], carrots, persimmons, pumpkins (by DPV), [46], *Vaccinium meridionale* swart/Agraz (by CV) [81], hops (Barth-Haas, Hallertau, Columbus, and Cascade) and malted barley samples (Chateau Cristal, Pilsen, Chateau Munich, and Wheat), sour cherry, pomegranate, plum, prickly pear (cactus fruit), date, and nectarine (by CV) [82], etc.

### 4.2. AOC Determination of the Plant’s Extract

Similar to the trend observed in the case of the fruit extracts, the technique of CV was found to be most effectively utilized for investigating the antioxidants of the plant’s extract as well [83]. However, the utilization of other techniques of DPV and SWV was found to be more abundant in comparison to the fruit extracts [84]. Khiya et al. [83] determined the AOC, TPC, condensed tannin content (TCT), and TAC of the *Salvia officinalis* by using the following assays/techniques of FRAP, DPPH, phosphomolybdate reduction, and CV. The extract acquired from the Boulemane region exhibited the highest value of total phenolic content (176 mgGAE/g), while the extract acquired from Khenifra region exhibited the highest TCT values (162.53 mgEC/g). In the case of TAC (highest value: 108 mgGAE/g), the aqueous fraction of the *S. officinalis* leaves acquired from the Boulemane region exhibited the best results. However, the CV results exhibited different results in comparison to other assays, as the best AOC results (288.8 mgGAE/g) were found in the case of the hydro-methanolic extract of the particular plant. Interestingly, the authors also highlighted the fact that the CV can only be utilized to specify the generalized type of phytochemicals; the technique of HPLC is still required for the documentation/characterization of individual molecules present in the understudy extract [60]. The authors also performed the correlation studies and concluded that the phenolic components (rather than flavonoid compounds) were responsible for AOC. This study highlights that every assay/technique has its limitation and the selection of the type of extracting medium (such as aqueous, methanoic, ethanoic or hydro-methanoic, etc.) should be considered beforehand for selecting the technique used for measuring AOC.

Amamra et al. [85] investigated the AOC values for *Thymus vulgaris* by utilizing spectroscopic assays and electrochemical techniques. The authors investigated butanol, ethyl acetate, aqueous, chloroform, petroleum ether, and methanolic extracts and revealed that all the extracts contained phenolics, flavonoids and tannins components. In terms of CV, the methanolic extract exhibited the best results with a correlation coefficient of 0.964 and 0.989 for gallic acid equivalent (GAE) and ascorbic acid equivalent, respectively, with the other assays (DPHH, Iron chelation assay, and 2,2-azinobis-(3-ethylbenzothiazoline-6-sulfonic acid)-radical cation (ABTS^+^) assay) for phenolic contents. Chochevska et al. [86] presented a comparison of the AOC values for the homemade-fruit vinegar (HMV) and commercial vinegar by using the technique of CV and exhibited that all the samples of HMV (acquired from the fermentation of the fruits of blueberry (*Vaccinium myrtillus*); apple (*Malus domestica*), raspberry (*Rubus idaeus*), rose hip (*Rosa canina*), blackberry (*Rubus fruticosus*), and persimmon (*Diospyros kaki*)) exhibited at least ten times more AOC values in comparison to the commercial vinegar samples. Fuentes et al. [87] used the technique of the chronoamperometry and chronopotensiometry techniques as a means to detect the AOC in the extracts of Aliso (*Alnusacuminate*) plant. Table 3 summarizes the critical remarks observed to be associated with the electrochemical techniques utilized for the detection of AOC.

## 5. Conclusions

This recent literature review presents critical insights into the use of the electrochemical methodologies as a tool to detect the AOC values of the plant and fruit extracts. The techniques of CV, DPV, SWV, and AM were explored for achieving this purpose. It was found that the CV is the most extensively exploited technique among all other electrochemical techniques for the detection of the AOC in numerous extract-based samples. In most studies, the electrochemical methodologies were used as an additional tool alongside other conventional (spectroscopic and chromatographic) techniques for the AOC determination, which is a welcome approach as the literature survey indicates that a contradiction still exists regarding the documented values of correlation of these electrochemical techniques with other technique. Furthermore, the AMM technique is far less explored in comparison to all other techniques, indicating that further research is still needed to explore the full potential/advantage of the electrochemical techniques for the determination of AOC. The nature of phytochemicals was also found to be critical as the AOC values of the extract containing the high molecular weight antioxidants cannot be found by using the electrochemical techniques.

## 6. Future Perspectives

Innovation in every field needs a multiprobe approach. The use of simulations and data-driven techniques can speed up the development in the investigation of the antioxidant potential of plant/fruit extracts. The density functional theory can be used to study the antioxidant potential of molecules in plant/fruit extracts [92,93,94]. The information from the theoretical analysis can help in selecting methods for experimental work. Recently, the use of machine learning has been increasing in biological and material science [95,96]. The machine learning models also can be used to predict the antioxidant potential of plants/fruits. It will shorten the discovery of efficient antioxidants; it is easy and more cost-effective way.

Following future perspectives can be observed regarding the use of the electrochemical methodologies for AOC values.

- The electrochemical techniques should be applied in conjugation with the chromatographic technique as the electrochemical techniques were still not able to identify the phytochemicals present in the extract.
- During the study of the correlation of the electrochemical techniques with the conventional methodologies, the fact that the electrochemical techniques are used to measure the TAC rather than measuring the particular antioxidants for the particular reaction should not be ignored. The cumulative antioxidant potential should be considered to be a preferable approach in determining the selective antioxidants via particular reactions.
- The flow injection analysis alongside the stationary electrochemical techniques should also be performed for studying the AOC potential of the extract.

## Figures and Tables

**Table 1:** Conventional assays/methods utilized for the determination of AC.

Classification	Assays	Mechanism Involved	Main Protocol	Advantages/Disadvantages	Detection Technique	Ref.
***Chemical-Based Assays***						
***ROS/free radical-based scavenging assay***	TEAC/ABTS assay	ET mechanism and HAT mechanism(Mixed mechanisms)	The AOC is estimated by studying the color diminishing capability of the sample. The ABTS^+^ radicals are colored and generated in the medium by the interaction of ABTS with K_2_S_2_O_8_. The antioxidant reaction with this organic radical provides the information regarding the AOC of the sample	***Advantages*** Rapid. Facile. Natural and synthetic antioxidant samples are studied. Assay can be applied over large pH ranges. Hydrophilic and hydrophobic antioxidants can be studied. ***Disadvantages*** Precision is moderate. The result of the assay depends upon the reaction time, which lowers the chance of comparative analysis. The steric hindrance and molecular size of the ABTS^+^ radical make it difficult for the antioxidants present in the medium to interact with this radical.	Calorimetry. Spectroscopy (decrease in the absorbance value at 743 nm).	[16]
	ORAC assay		The radical chain-breaking potential of the antioxidants present in the sample by blocking the peroxy radical generation of AAPH. The peroxy radicals react with the fluorescent probe to generate the non-fluorescent product.	***Advantages*** Food-based samples involving the peroxy radicals (lipid oxidation) can be properly assessed by this assay. Rapid. Can be automated. ***Disadvantages*** A competitive technique where the antioxidant and fluorescent probe compete for scavenging the peroxy radicals. Use of fluorescent probe (ß-PE) can cause false ORAC readings. For studying the AOC for hydrophilic and hydrophobic antioxidants, the modification of ORAC assays is required.	Fluorimetry (decrease in the fluorescence intensity area under the curve of fluorescence intensity versus time graph). Quantification of the non-fluorescent product generated.	[17]
	TRAP assay		The luminol-enhanced chemiluminescence owing to the presence of the luminol-derived radicals (generated due to the AAPH thermal decomposition) is quenched by the antioxidants present in the sample.	***Advantages*** Rapid. Change can be visually observed. ***Disadvantages*** Laborious and complex methodology to perform. The antioxidants that do not have a clear lag phase cannot be detected by using TRAP assay. Low reproducibility.	Quenching of chemiluminescence. The duration of the lag phase is utilized for the quantification of the antioxidant.	[18]
	ß-Carotene bleaching assay		Aqueous emulsion of ß-carotene and linoleic acid generates the free radical in the medium owing to the spontaneous oxidation of the fatty acids. The sample is decolorized owing to the presence of antioxidants in the medium.	***Advantages*** Rapid. Change can be visually observed. Can screen both the hydrophobic and hydrophilic antioxidants. ***Disadvantages*** ß-Carotene is sensitive to temperature and pH change which causes the errors in reading. Low reproducibility.	Calorimetry. Spectroscopy (decrease in the absorbance at 470 nm).	[19]
	DPPH assay		The AOC is estimated by studying the decrease in the absorbance value at 517 nm associated with the DPPH^·^ radical. The antioxidant reaction with this organic radical lowers the concentration of the radical in the medium that expresses itself by the reduction in the absorbance intensity at the characteristic wavelength of the radical.	***Advantages*** Rapid. Facile. Low cost. ***Disadvantages*** Lacking standardization. The reaction parameters (including pH, solvent, dissolved oxygen, exposure to light radiations, etc.) affect the AOC values. This kinetic fact is generally ignored. The steric hindrance and molecular size of the DPPH^·^ radical make it difficult for the antioxidants present in the medium to interact with this radical. DPPH is not favorable for ranking the antioxidants owing to its dependence on numerous uncontrollable factors.	Calorimetry. Spectroscopy (decrease in the absorbance at 517 nm).	[16]
	TOSC assay		The oxidation of KMBA results in the formation of ethylene by ROS. Presence of antioxidants inhibits the formation of ethylene.	***Advantages*** Rapid. Facile. Low cost ***Disadvantages*** Low accuracy.	Headspace gas chromatography for the determination of ethylene.	[20]
***Redox potential based assay (non-radical based assays)***	CUPRAC assay	ET mechanism	Potential of antioxidants to reduce the Cu^2+^ ions into the Cu^1+^ ions is utilized as a means to estimate the AOC. The Cu^1+^ ions form a complex with neocuproine or bathocuproine, which is detected by spectrophotometric analysis.	***Advantages*** Rapid. Facile. Hydrophilic and lyophilic antioxidants can be studied. ***Disadvantages*** Require the specific pH. Variation in the pH makes this method ineffective as the complex formation is hindered. CUPRAC cannot measure the HAT mechanism and therefore, it cannot be utilized to study the intracellular fluids and the human blood plasma/serum.	Calorimetry. Spectroscopy (appearance of absorption band at 450 nm and 490 nm in case of neocuproine and bathocuproine, respectively).	[21]
	FRAP assay		Potential of antioxidants to reduce the [Fe^3+^-(TPTZ)_2_]^3+^ complex into the [Fe^2+^-(TPTZ)_2_]^2+^ is utilized as a means to estimate the AOC.	***Advantages*** Rapid. Facile. ***Disadvantages*** Require the acidic medium. Variation in the pH makes this method ineffective as the iron complex undergoes precipitation. FRAP cannot measure the HAT mechanism and therefore, it cannot be utilized to study the intracellular fluids and the human blood plasma/serum.	Calorimetry Spectroscopy (increase in the absorption values at 593 nm).	[21]
	TPC or FCR assay		The Folin–Ciocalteu reagent in a strong basic environment generates the phenolate anions, which are reduced by the phenolic antioxidants present in the sample.	***Advantages*** Rapid. TPC/FCR assay determines the total antioxidant assay. Operationally simple. Reproducible. ***Disadvantages*** Non-phenolic compounds can interfere with the Folin–Ciocalteu reagent and cause error in the readings. Strong basic environment is required.	Colorimetry Spectroscopy (appearance of blue-colored chromophore having maximum wavelength values in the range of 620–765 nm).	[22]
	Metal chelation capacity		Chelation effects of antioxidants for the quenching of the metal ions (ferrous ions) are used as a means to measure the AOC values.	***Advantage*** Utilized to study the AOC value of the secondary antioxidants. The test provides the accurate assessment of the antioxidant potential of compounds during lipid peroxidation. ***Disadvantages*** Standardization of the protocol is required.	Spectroscopy (decrease in absorbance intensity of Fe^2+^-ferrozinen at 562 nm).	[23]
***Biochemical based assay***						
***In vivo* assay**	LDL assay	ET/HAT mechanisms	The oxidation capacity of LDL lipid peroxidation is reduced owing to the presence of antioxidants.	***Advantage*** Utilized to study the AOC value of the secondary antioxidants. The test provides an accurate assessment of the antioxidant potential of compounds during lipid peroxidation. Endogenous antioxidants can be studied by using the LDL assay. ***Disadvantages*** Standardization of the protocol is required. Mechanism involved in the quenching requires further exploration.	Chemiluminescent (radiations emitted by the oxidative products). Spectroscopy (formation of diene conjugates at 234 nm).	[24]
	TBARS assay		The unsaturated lipid degradation results in the formation of MDA in the presence of ROS, and this MDA interact with TBA to produce [MDA-(TBA)_2_] complex. The presence of antioxidants reduces the formation of this complex, which is used as a tool to measure the AOC of the antioxidant.	***Advantage*** Utilized to study the AOC value of the secondary antioxidants. The test provides an accurate assessment of the antioxidant potential of compounds during lipid peroxidation. ***Disadvantages*** It is a pH-dependent procedure. Acidic pH and temperature values of 100 °C are essential for carrying out the reaction.	Calorimetry Spectroscopy (Reduction in the [MDA-(TBA)_2_] complex presents the absorption band at 532 nm).	[25]
	CAA assay		Within the cell medium, the DCFH-DA is deacylated by cellular enzymes to generate DCFH within the cells. The peroxy radicals formed from AAPH easily cross the cellular membrane and convert the DCFH into fluorescent DCF. The presence of the antioxidants reduces the DCF generation.	***Advantage*** The performance of the test within the cell medium makes this test the best representative test for studying antioxidants present within the cell. Dietary supplements, food samples, beverages, and other biogenic samples can be studied by using this assay. ***Disadvantages*** It is a complex test with intercalated mechanisms. The results acquired from the CAA assay do not correlate well with the chemical assays.	Fluorimetry (Decrease in the fluorescence intensity).	[26]

Abbreviations: Reactive oxygen species (ROS), Trolox equivalent antioxidant activity (TEAC), 2,2′-azino-bis (3-ethylbenzothiazoline-6-sulphonic acid) (ABTS), 2,2′-azino-bis (3-ethylbenzothiazoline-6-sulphonate) radical cation (ABTS^+^), 2,2-diphenyl-1-picrylhydrazyl (DPPH), 2,2-diphenyl-1-picrylhydrazyl radical (DPPH**^·^**), total peroxyl radical trapping antioxidant parameter (TRAP), Oxygen radical antioxidant capacity (ORAC), total oxyradicals scavenging capacity (TOSC), Ferric reducing antioxidant power (FRAP), Cupric reducing antioxidant capacity (CUPRAC), Cyclic voltammetry (CV), Differential pulse voltammetry (DPV), Square wave voltammetry (SWV), low density lipoproteins (LDL), thiobarbituric acid reactive substances (TBARS), cellular antioxidant activity (CAA), Electron transfer (ET), Hydrogen atom transfer (HAT), antioxidant capacity (AOC), Potassium persulfate (K_2_S_2_O_8_), 2,2′-azo bis(2-methylpropionamidine) hydrochloride (AAPH), ß-phycoerythrin (ß-PE), α-keto-γ-methiolbutyric acid (KMBA), 2,4,6-tripyridyl-s-triazine complex [Fe^3+^-(TPTZ)_2_]^3+^, Cupric ion (Cu^2+^), Cuprous ion (Cu^1+^), Folin–Ciocalteu reducing assay (FCR), Malondialdehyde (MDA), thiobarbituric acid (TBA), 2′,7′-dichlorofluorescin diacetate (DCFH-DA), 2′,7′-dichlorofluorescin (DCFH), dicholorofluorescin (DCF).

**Table 2:** Some analytical methods applied to determine the antioxidant and total antioxidant capacities..

Detection Method	Samples Analysed for Antioxidant Capacity	Nature of the Sample	Detection Assay	Application of Antioxidant	Ref.
Spectroscopic methods	Red *propolis* extracts	Natural	Multivariate analysis (determining phenolic compounds and their bioactive properties) DPPH assay The reducing power assay β-carotene assay	Several types of biological activities were documented attributed to its chemical constituents	[29]
	Aqueous and methanolic extracts from *A. fragrans* leaves (Two dihydrochalcone glycosides named confusoside and vacciniifolin)	Natural Extract	DPPH assay ABTS assay FRAP assay	A bioactive functional plant and the leaves of *A. fragrans* could be used as a potential source of natural antioxidants for food and pharmaceutical applications	[30]
	Co (II) and Fe (II) complexes of Schiff base	Synthetic	In vitro assays FRAP assay CUPRAC assay ABTS assay DPPH assay Enzyme inhibition studies.	Antioxidant activities and enzyme inhibition determination were carried out. The best inhibitor complex for CA I and II isoenzymes was the Fe(II) complex with *K_i_* values of 52.83 ± 11.52 and 63.34 ± 8.88, respectively	[31]
	Caffeic acid grafted chitosan	Synthetic	DPPH assay	A novel dual-functional stabilizer for food-grade emulsions and additive antioxidant property	[32]
	γ-alumina nanoparticles	Synthetic	DPPH assay Computational methods (DFT-D studies)	Radical scavenging performance and antioxidant activity	[33]
Chromatographic Methods	*Achillea Arabica* Contents of phenolics, including caffeoylquinic acid derivatives, quercetin, luteolin, apigenin and some of their derivatives	Natural	HPLC-PDA fingerprint analysis ABTS assay FRAP assays.	It can be incorporated in food and pharmaceutical applications	[34]
	Essential antioxidant algal pigments present in marine algae (*S. vulgare*, *C. barbata* brown, and *F. petiolata* green algae species)	Natural	CUPRAC assay ABTS assay HPLC	Potentially rich sources of valuable nutrients and the favorable choice in many different industrial fields: hydrocolloid extraction, fertilizers, extracts for cosmetics and pharmaceuticals, biofuels, bioplastics, and wastewater treatment	[35]
	Flavone triglycosides identified in sugarcane molasses (Chrysoeriol-C-glycosides such as scoparin	Natural	ABTS assay CAA assays LCMSs	Can be used as potential source of polyphenols that can be beneficial to health	[36]
	Mulberry leaf (Prenylated phenolics a, moracin N)	Natural	DPPH assay CAA assay HPLC UHPLC	Multiple health benefits	[37]
Electrochemical methods	Citrus peel residues (*flavedo*)	Natural	CUPRAC assay DPPH assay	Food, pharmaceutical and cosmetic industries	[38]
	Plant tea samples such as green tea, lime, and coral moss	Natural	Voltammetry	Nutritional science	[39]
	quinones: Mansanone-D, Mansanone-H, Sissoidenone and 4-methoxy dalbergione isolated from *Dalbergia sissoides*	Natural	CV	Antioxidant activity and strong interaction with DNA	[40]

Abbreviations: 2,2′-azino-bis (3-ethylbenzothiazoline-6-sulphonic acid) (ABTS), 2,2-diphenyl-1-picrylhydrazyl (DPPH), Oxygen radical antioxidant capacity (ORAC), total oxyradicals scavenging capacity (TOSC), Ferric-reducing antioxidant power (FRAP), Cupric reducing antioxidant capacity (CUPRAC), Cyclic voltammetry (CV), Dispersion corrected density functional theory (DFT-D), High-performance liquid chromatography (HPLC), High-performance liquid chromatography with photodiode array (HPLC-PDA), Liquid chromatography with mass spectroscopy (LCMS), Ultra-performance liquid chromatography (UHPLC), cyclic voltammetry (CV).

**Table 3:** Summary of the case studies documenting the AC values and other parameters calculated by using the electrochemical techniques.

Studied Extract	Extract Type	Conventional Techniques	Electrochemical Techniques	Electrodes	Parameter Identified	Remarks	Ref.
Zespri^®^ Sweet Green kiwifruit (*Actinidia chinensis x Actinidia deliciosa*)	Flesh and skin extract of the kiwi fruit	For phenolic contents: TPC analysis TFC analysis TAC analysis ABTS assay DPPH assay FRAP assay	CV coupled with the HPLC	Reference Electrode: Ag/AgCl electrode Working Electrode: Glassy carbon electrode	Identification of phenolic contents by comparing them with the phenolic standards. Identified phenolic components: Catechin Gallic acid Protocatechuic acid Vanillic acid Caffeic acid Syringic acid Epicatechin Epigallocatechin gallate p-Coumaric acid Rutin Ferulic acid Epicatechin gallate Quercetin trans-Cinnamic acid The reducing potential and AOC of these compounds were studied by using the parameters of E_pa_, I_pa_ and Q_500_.	Among the fourteen identified phenolic components, CV was able to identify the nine antioxidant molecules present in the extract. Moreover, CV was identified to be the better technique in comparison to the spectroscopic and antioxidants assays as it records the total AOC values in comparison to the particular mechanism (scavenging, ability to reduce metal, or lipid peroxidation inhibition. One drawback that was observed in case of CV technique was that the ascorbic acid could not be studied through this, while other techniques detected it in the phytochemical profiling.	[50]
Zespri^®^ SunGold Kiwifruit (*Actinidia chinensis*)							
Hayward commercial fruit							
*Vitis vinifera* L. (Grape pomace)	Grape pomace (seed, skin and stem extract)	TPC analysis TBARS assay	CV	Three-electrode system Reference system: Ag/AgCl electrodeWorking electrode: Glassy carbon electrode Auxiliary electrode: Platinum wire	Identification of electrochemical phenolic compounds: (3′, 4′-dihydroxyl moiety of phenolic compounds). The Q_500_ parameter was used to identify the total phenolic content of the extract. The I_pc_, I_pa_, E_pa_, E_pc_, and E_mid_ parameters are utilized to investigate the following observables: Total AOC of the extracts Byproducts after the antioxidant reaction Concentration of antioxidantsElectrochemical mechanism	Individual phenols are not detected. Correlation between the TBARS and CV was studied by Pearson’s correlation.	[80]
*Greigia Sphacelata*	Fruit	ORAC test	DPV	Three-electrode system Reference system: Ag/AgCl electrodeWorking electrode: Glassy carbon electrode Auxiliary electrode: Platinum wire	Current, potential and area under the current-potential curve are the main Electrochemical parameters utilized to investigate the AOC potential of the sample.	Despite differences in the operating mechanism of the utilized assays of ORAC assay (fluorescent method) and DPV (an electrochemical method), Pearson’s correlation test revealed that both methods exhibited a correlation value of 0.969 with each other. The authors also reported that the understudy sample has very low antioxidant activity values.	[88]
*Solanum nigrum*	Black nightshade fruit	TPC analysis TFC analysis TAC analysis DPPH assay FRAP assay	CV	Three-electrode system Reference system: Hg/Hg_2_Cl_2_ electrode Working electrode: Glassy carbon electrode Auxiliary electrode: Platinum wire	E_pa_ and I_pa_ values were used to estimate the AOC values of the fruit extract. The higher I_pa_ values were co-related with, the higher antioxidant activity. The shape of the voltammogram (i.e., absence of the cathodic peak) was used to obtain information regarding the irreversibility of the phenolic components present in the extract.	Superoxide radical scavenging assay was utilized as a means to study the AOC values by using the CV technique, indicating that CV can be used to investigate the ability of the extract to quench the superoxide radical. Ethyl acetate extract of fruit expressed better AOC and scavenging results in comparison to chloroform and n-butanol extract of the fruit.	[89]
*Phoenix dactylifera.* L.	Date palm fruit	TPC analysis TFC analysis DPPH assay FRAP assay	CV	Three-electrode system Reference system: Hg/Hg_2_Cl_2_ electrode Working electrode: Glassy carbon electrode Auxiliary electrode: Platinum wire	Reduction in the I_pa_ values associated with the superoxide radical was used as a representation tool indicating the decrease in the concentration of radical owing to the presence of phytochemicals in the extract. The shape of voltammogram is used for studying the irreversibility of the phytochemicals involved.	Authors observed that spectrophotometer suffers from the drawback that phytochemicals exhibit the peaks in the same range and, consequently, are not properly identified by spectroscopic techniques. The electrochemical method removes this problem.	[62]
*Bunchosia glandulifera*	Seed, bark, leaf and root extract	TPC analysis β-Carotene test Ascorbic acid test DPPH assay FRAP assay ABTS assay	CV DPV	Three-electrode system Reference system: Ag/AgCl electrode Working electrode: Glassy carbon electrode Auxiliary electrode: Platinum wire	Current, potential and area under the current-potential curve are the main electrochemical parameters utilized to investigate the AOC potential of the sample	The solvent of DMSO is noxious and its use as a solvent should not be encouraged. However, the study presented an excellent comparison between electrochemical methodologies and revealed that the CV showed better linearity in the case of the DPV. Furthermore, the authors found the AOC value of the seed extract presented the best results among all other extracts.	[90]
*Punica granatum* L.	Pomegranate peels	TPC analysis TFC analysis TCT analysis DPPH assay FRAP assay	CV	Three-electrode system Reference system: Ag/AgCl electrode Working electrode: Glassy carbon electrode Auxiliary electrode:Platinum wire	Anodic current, anodic potential and area under the current-potential curve are the main electrochemical parameters utilized to investigate the AOC potential of the sample.	The extraction of the phenolic components was performed by using the response surface methodology (RSM). The authors identified that the AOC potential of the five medicinal plants exhibited almost 36 times lower efficacy in comparison to the ascorbic acid-based test, indicating that the CV is not suitable for measuring the AOC value for the extracts containing the high molecular weight antioxidants.	[91]
*Argania spinosa* L.	Argan hull						
*Corrigiola telephiifolia Pourr.*	Serghina						
*Prunus dulcis*	Almond						
*Rubia tinctorum* L.	Madder root						

Abbreviations: Total Phenolic Content (TPC), Total Flavonoid Content (TFC), Total Flavanol Content (TAC), 2,2-azinobis-(3-ethylbenzothiazoline-6-sulfonic acid)-diammonium salt (ABTS), 2,2-diphenyl-1-picrylhydrazyl (DPPH), ferric reducing ability of plasma (FRAP), High-Performance Liquid Chromatography (HPLC), Cyclic voltammetry (CV), silver/silver chloride (Ag/AgCl), Oxygen radical absorption capacity (ORAC), Dimethyl sulfoxide (DMSO).
